# Antifungal prophylaxis for prevention of COVID-19-associated pulmonary aspergillosis in critically ill patients: an observational study

**DOI:** 10.1186/s13054-021-03753-9

**Published:** 2021-09-15

**Authors:** Stefan Hatzl, Alexander C. Reisinger, Florian Posch, Juergen Prattes, Martin Stradner, Stefan Pilz, Philipp Eller, Michael Schoerghuber, Wolfgang Toller, Gregor Gorkiewicz, Philipp Metnitz, Martin Rief, Florian Prüller, Alexander R. Rosenkranz, Thomas Valentin, Robert Krause, Martin Hoenigl, Gernot Schilcher

**Affiliations:** 1 Intensive Care Unit, Department of Internal Medicine, Medical University of Graz, Graz, Austria; 2 Division of Haematology, Department of Internal Medicine, Medical University of Graz, Graz, Austria; 3 Division of Oncology, Department of Internal Medicine, Medical University of Graz, Graz, Austria; 4 Division of Infectious Diseases, Department of Internal Medicine, Medical University of Graz, Graz, Austria; 5 Division of Rheumatology and Immunology, Department of Internal Medicine, Medical University of Graz, Graz, Austria; 6 Division of Endocrinology and Diabetology, Department of Internal Medicine, Medical University of Graz, Graz, Austria; 7 Department of Anaesthesiology and Intensive Care Medicine, Medical University Graz, Graz, Austria; 8 Institute of Pathology, Medical University of Graz, Graz, Austria; 9 Clinical Institute for Medical and Chemical Laboratory Diagnostics, Medical University of Graz, Graz, Austria; 10 Division of Nephrology, Department of Internal Medicine, Medical University of Graz, Graz, Austria; 11 Division of Infectious Diseases, University of California San Diego, San Diego, USA

**Keywords:** ICU, CAPA, COVID-19-associated aspergillosis, Posaconazole, Mould prophylaxis

## Abstract

**Background:**

Coronavirus disease 19 (COVID-19)-associated pulmonary aspergillosis (CAPA) emerged as important fungal complications in patients with COVID-19-associated severe acute respiratory failure (ARF). Whether mould active antifungal prophylaxis (MAFP) can prevent CAPA remains elusive so far.

**Methods:**

In this observational study, we included all consecutive patients admitted to intensive care units with COVID-19-associated ARF between September 1, 2020, and May 1, 2021. We compared patients with versus without antifungal prophylaxis with respect to CAPA incidence (primary outcome) and mortality (secondary outcome). Propensity score adjustment was performed to account for any imbalances in baseline characteristics. CAPA cases were classified according to European Confederation of Medical Mycology (ECMM)/International Society of Human and Animal Mycoses (ISHAM) consensus criteria.

**Results:**

We included 132 patients, of whom 75 (57%) received antifungal prophylaxis (98% posaconazole). Ten CAPA cases were diagnosed, after a median of 6 days following ICU admission. Of those, 9 CAPA cases were recorded in the non-prophylaxis group and one in the prophylaxis group, respectively. However, no difference in 30-day ICU mortality could be observed. Thirty-day CAPA incidence estimates were 1.4% (95% CI 0.2–9.7) in the MAFP group and 17.5% (95% CI 9.6–31.4) in the group without MAFP (*p* = 0.002). The respective subdistributional hazard ratio (sHR) for CAPA incidence comparing the MAFP versus no MAFP group was of 0.08 (95% CI 0.01–0.63; *p* = 0.017).

**Conclusion:**

In ICU patients with COVID-19 ARF, antifungal prophylaxis was associated with significantly reduced CAPA incidence, but this did not translate into improved survival. Randomized controlled trials are warranted to evaluate the efficacy and safety of MAFP with respect to CAPA incidence and clinical outcomes.

**Supplementary Information:**

The online version contains supplementary material available at 10.1186/s13054-021-03753-9.

## Introduction

Severe acute respiratory syndrome coronavirus 2 (SARS-CoV-2) disease 2019 (COVID-19)-associated acute respiratory failure contributes to a highly permissive inflammatory environment. This in turn favours fungal pathogenesis due to the release of danger-associated molecular patterns and collateral effects of host recognition pathways required for the activation of antiviral immunity [1]. In this context, COVID-19-associated pulmonary aspergillosis (CAPA) has been emerging as an important fungal complication of COVID-19 [1], affecting an average of 3.1% (range 0.7–7.7%) [2–5] of patients hospitalized with COVID-19, 8.9% (range 2.5–39%) of patients admitted to the intensive care unit (ICU) [3, 6–11], and an average of 20.1% (range 3.2–38%) of those requiring invasive ventilation [6, 7, 12–14].

Diagnosis of CAPA is challenging in patients with COVID-19-associated ARDS, as clinical picture and radiological findings of CAPA resemble those of severe COVID-19 [15, 16], and blood tests lack sensitivity due to the primarily airway invasive growth of *Aspergillus* in non-neutropenic patients [15–17]. Testing of bronchoalveolar lavage (BAL) with fungal culture, galactomannan (GM), *Aspergillus* polymerase chain reaction (PCR), or the *Aspergillus* GM lateral flow assay (LFA) is therefore preferred [18, 19], but due to the presumed risk of COVID-19 transmission through bronchoscopies, sampling of the primary infection site is still not performed consistently across ICUs.

The high prevalence rates of CAPA in critically ill patients requiring invasive ventilation together with the difficulties in diagnosis and the devastating overall mortality rates of over 50% [2–4, 6, 9–12, 14, 15, 20, 21] could justify clinical trials evaluating antifungal prophylaxis in COVID-19 patients with acute respiratory failure. One retrospective single-centre case series from Belgium has reported the successful use of prophylaxis in terms of CAPA case reduction with inhaled liposomal Amphotericin B in a cohort of ICU patients with severe COVID-19 [6]; however, studies evaluating systemic antifungal prophylaxis are lacking.

The objective of this observational single-centre study was to evaluate the effectiveness of mould-active antifungal prophylaxis in preventing CAPA in critical care patients with COVID-19-associated acute respiratory failure. Secondary objectives included the evaluation of a potential survival benefit associated with antifungal prophylaxis as well as the impact of CAPA on overall survival.

## Methods

### Study cohort

We performed an observational study, enrolling all consecutive adult SARS-CoV-2 polymerase chain reaction (SARS-CoV-2 PCR) positive patients to our ICUs at the Department of Internal Medicine, Medical University of Graz, Austria (later referred to as ICU 1), or the Department of Anaesthesiology, Medical University of Graz, Austria (later referred to as ICU 2), between September 1, 2020, and May 1, 2021.

All patient data were uniformly collected as described previously [22, 23]. Therefore, laboratory, clinical and radiology data were extracted from our in-house electronic healthcare database system and inserted in a predefined electronic case report form (eCRF) using REDCap electronic data capture [24, 25]. For classification of CAPA, the 2020 European Confederation of Medical Mycology (ECMM)/International Society of Human and Animal Mycoses (ISHAM) consensus criteria were used [16]. According to these criteria, patients were categorized as either proven, probable or possible pulmonary and/or tracheobronchial CAPA, or no evidence for CAPA. In those receiving antifungal prophylaxis, CAPA cases were further classified according to the ECMM/MSG definitions for breakthrough infections [26]. To reduce confirmation bias, CAPA classification was made by two infectious disease specialists who were blinded against the baseline characteristics and the administration of mould active antifungal prophylaxis.

**Fig. 1:**
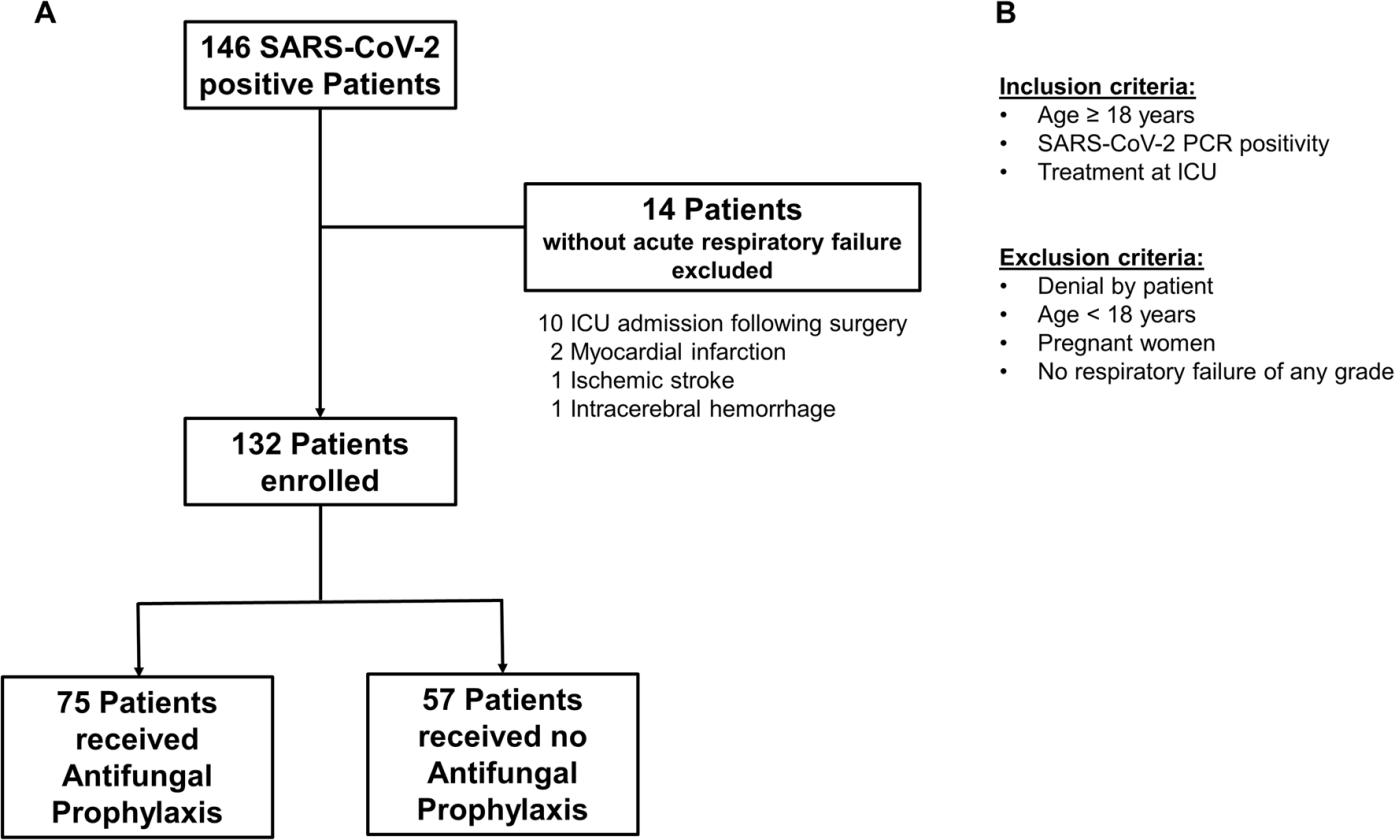
Full trial protocol and flow diagram. **A** 146 patients were initially enrolled. 14 patients were admitted for other reason than acute respiratory failure (10 post-surgical, 2 myocardial infarctions, 1 ischemic stroke, 1 intracranial haemorrhage). Therefore, 132 patients were included in the analyses, whereof 75 received antifungal prophylaxis and 57 did not receive antifungal prophylaxis. **B** Inclusion and exclusion criteria of the study. COVID-19 coronavirus disease 19; SARS-CoV-2 severe acute respiratory syndrome corona virus 2; PCR polymerase chain reaction; ICU intensive care unit

Data analysis was performed after exclusion of fourteen patients who did not meet the predefined inclusion criteria or met a predefined exclusion criterion after initial screening (Fig. 1).

The research project was approved by the local institutional review board (EC #32–296 ex 19/20).

### Antifungal prophylaxis group and control group

Assignment to antifungal prophylaxis was informed by our in-house recommendation, which recommended antifungal prophylaxis with posaconazole or a similar mould active antifungal drug for every COVID-19 patient with acute respiratory failure admitted to the ICU. This recommendation was based on our experience with COVID-19-associated mould infections occurring before September 2020 [16, 27, 28] and on frequent fungal resistance testing within our centre which revealed no case of azol resistance in *Aspergillus spp. to date*. This recommendation was first implemented at ICU 1 and later at ICU 2. However, the decision of administration of mould active antifungal prophylaxis largely relays on the treating intensivist allowing us to compare outcomes of patients with and without antifungal prophylaxis who were otherwise treated according to same medical standards. Treated patients and control patients were distributed among both intensive care units.

### Statistical analysis

All statistical analyses were performed using Stata (Windows version 16.1; Stata Corp., Houston, TX, USA) and R 4.0.5 (https://www.r-project.org/) and followed an a priori specified analysis plan (Additional file 1: Figure S1). The distribution of baseline variables between the antifungal prophylaxis and no antifungal prophylaxis group was evaluated using rank-sum tests, χ^2^-tests, and Fisher’s exact tests, as appropriate. The magnitude of these differences was quantified with standardized mean differences (SMDs), and SMDs ≥ 0.31 were considered indicative of a potentially relevant magnitude of difference. To account for differences in covariates between the groups with and without antifungal prophylaxis, we used propensity score analyses. We obtained the propensity score *e* from a multivariable logistic regression model with antifungal prophylaxis group as the outcome variable. For this model, we pre-specified a fixed number of maximum 10 predictor variables, in order not to have less than 3 events per predictor variable. In detail, the model was developed by initially including all variables with a p for difference between the two groups of ≤ 0.16 and/or a SMD ≥ 0.31. The propensity score *e* was then transformed into an inverse-probability-of-treatment-weight (IPTW) according to the average treatment effect principle, i.e. $=\frac{(Antifungal Prophylaxis)}{e}+\frac{1-(Antifungal Prophylaxis)}{1-e}$, where antifungal prophylaxis denotes the treatment assignment[29]. For balance diagnostics, we then re-estimated SMDs and p-values for difference between the two treatment groups with the IPTW-weighted data [30, 31]. For this propensity score model, missing data of ten variables (body mass index, paO_2_/FiO_2_, positive end expiratory pressure, lactate, interleukin 6, C-reactive protein, ferritin, high sensitive troponin T, D-Dimer, absolute lymphocyte count) were imputed using a chained equations algorithm [32]. Between-group tests for differences were re-performed after weighting with the IPTW.

Sensitivity analyses included a trimmed IPTW (i.e. excluding patients with an IPTW ≤ the 1st and ≥ the 99th percentile of its distribution).

The primary outcome of the study, occurrence of CAPA within 30-days of ICU admission, was defined as the time interval from ICU admission to the time point when the first positive sample for CAPA was obtained or the censoring date which was set the latest at 30 days from ICU admission. Survival time was inflated by one day in patients who died at the day of ICU admission (*n* = 2). The secondary outcome of the study, 30-day ICU overall survival (OS), was defined as the time interval from ICU admission to death-from-any-cause or the censoring date when being still alive 30 days after ICU admission.

In addition, we also analysed 90-day ICU overall survival to evaluate long-term ICU survival.

Univariable unweighted and IPTW-weighted Cox proportional hazards models and Fine and Gray proportional sub-distribution hazards models were fitted for analysing the association between antifungal prophylaxis and time-to-event end points, respectively.

Univariable unweighted and IPTW-weighted risks of development of CAPA and death-from-any-cause were analysed with competing risk cumulative incidence estimators, Gray’s tests, and Fine and Gray proportional sub-distribution hazards models, respectively. Death-from-any-cause was considered as competing event of interest in the analyses of CAPA development risk [33].To eliminate immortal time bias in the analysis of the association of CAPA with ICU overall survival, the occurrence of CAPA was modelled as a time-dependent variable within Cox models. The association of CAPA with ICU survival was defined as co-secondary outcome. This was achieved by partitioning the follow-up time of patients who developed CAPA into times before and after CAPA diagnosis. For visual display of the association between CAPA and ICU overall survival outcomes, we performed a landmark analysis after 14 days of ICU admission. This landmark was chosen because most CAPA diagnosis happened in the first 2 weeks after ICU admission.

The full dataset and the main analysis code are available upon request by the first author.

In all analyses *p* values < 0.05 were considered statistically significant.

## Results

### Cohort description and baseline characteristics

Over the study period, 132 patients were admitted to the ICUs due to COVID-19-associated acute respiratory failure and therefore included in the final analysis (Fig. 1). Of those, 75 (57%) patients received mould active antifungal prophylaxis within the first 48 h after ICU admission according to our local standard, while the remaining 57 (43%) did not. At the time of ICU admission, the median age of patients was 65 years [25th–75th percentile: 55–75], and 48 (36%) patients were female. The investigated population had a median of two coexisting conditions [1–4] and a median time from SARS-CoV-2 PCR positivity to ICU admission of 3 days [0–6]. All patients included in this study showed an oxygen saturation of less than 88% while breathing ambient air prior to ICU admission and were classified as acute respiratory failure. Most patients exhibited severe acute respiratory failure as displayed by a median paO2/FiO2 ratio (Horowitz Index) of 95 [25th–75th percentile 75–146]. Seventy-two patients (56%) required invasive ventilation including veno-venous extracorporeal membrane oxygenation (vvECMO) (*n* = 13) and/or endotracheal intubation (*n* = 59). All patients in this study received systemic glucocorticoids as supportive treatment. Remdesivir was administered in 33 (40%) patients, convalescent plasma was given to 50 (38%) patients, whereas only one patient received tocilizumab (Table 1). During the first 30-days after ICU admission, we observed 10 CAPA cases, corresponding to 1-, 15-, and 30-day CAPA incidence of 0.8% (95% CI 0.1–5.2), 8.5% (4.6–15.2), and 8.5% (4.6–15.2), respectively, in the complete cohort (Additional file 2: Figure S2).

**Table 1:** Baseline characteristics of the study population—distribution at ICU admission

Variable	n (%miss)	Overall (*n* = 132)	Antifungal prophylaxis (*n* = 75)	No antifungal prophylaxis (*n* = 57)	*p*	*p*_*IPTW*_
**Demographic variables**						
Age (years)	132 (0%)	65 [55–75]	67 [55–74]	63 [56–75]	0.879	0.375
Female Gender	132 (0%)	48 (36%)	24 (32%)	24 (42%)	0.232	0.632
BMI (kg/m^2^)	127 (4%)	28.6 [25.4–33.2]	27.8 [24.5–32.8]	29.6 [25.9–33.7]	0.139	0.285
**Coexisting conditions**						
Number of coexisting conditions	132 (0%)	2 [1–4]	2 [1–3]	2 [1–4]	0.629	0.640
Hypertension	132 (0%)	93 (71%)	49 (65%)	44 (77%)	0.186	0.562
Diabetes	132 (0%)	31 (23%)	20 (27%)	11 (19%)	0.323	0.454
Atrial fibrillation	132 (0%)	22 (17%)	10 (13%)	12 (21%)	0.238	0.400
Coronary artery disease^$^	132 (0%)	21 (16%)	9 (12%)	12 (21%)	0.159	0.686
Congestive heart failure	132 (0%)	20 (15%)	9 (12%)	11 (19%)	0.247	0.763
Peripheral arterial disease	132 (0%)	17 (13%)	7 (9%)	10 (17%)	0.163	0.171
Thromboembolic disease	132 (0%)	19 (14%)	10 (13%)	9 (16%)	0.690	0.230
Chronic kidney disease	132 (0%)	35 (26%)	15 (20%)	20 (35%)	0.052	0.892
Dialysis	132 (0%)	11 (8%)	2 (3%)	9 (16%)	0.007	0.656
COPD	132 (0%)	16 (12%)	13 (17%)	3 (5%)	0.035	0.867
Asthma	132 (0%)	15 (11%)	9 (12%)	6 (11%)	0.792	0.702
Prior cancer in complete remission	132 (0%)	11 (8%)	6 (8%)	5 (8%)	0.874	0.917
Active malignancy	132 (0%)	9 (7%)	6 (8%)	3 (5%)	0.537	0.644
Dementia	132 (0%)	4 (3%)	2 (3%)	2 (4%)	0.780	0.844
Prior transplantation	132 (0%)	10 (8%)	7 (9%)	3 (5%)	0.381	0.821
Immunosuppression †	132 (0%)	17 (13%)	13 (17%)	4 (8%)	0.008	0.921
**ICU risk stratification**						
SOFA (points)	132 (0%)	5 [4–8]	4 [4–7]	5 [4–10]	0.057	0.987
paO_2_/FiO_2_	128 (3%)	95 [75–146]	90 [75–130]	107 [75–152]	0.302	0.749
PEEP (mmHg)—maximum	111 (16%)	11 [9–12]	10 [9–12]	11 [10–12]	0.599	0.464
Acute respiratory failure grade (inspired by ARDS *Berlin 2015* -classification)	132 (0%)	–	–	–	0.660	0.636
–severe		76 (58%)	46 (61%)	30 (54%)	–	
–moderate		45 (34%)	24 (32%)	21 (37%)	–	
–mild		10 (8%)	5 (7%)	6 (9%)	–	
Ventilation	132 (0%)	–	–	–	0.146	0.776
–Intubated		59 (45%)	35 (47%)	24 (42%)	–	
–vvECMO		13 (10%)	6 (8%)	7 (12%)	–	
–NIV		49 (37%)	31 (41%)	18 (32%)	–	
–HFNC		4 (3%)	2 (3%)	2 (3%)	–	
–Mask oxygen supply		7 (5%)	1 (1%)	6 (11%)	–	
Any invasive ventilation		72 (56%)	41 (55%)	31 (54%)	0.974	0.947
**Antimycotic substances**						
Duration form diagnosis to initiation (days)	75 (0%)	–	4 [1–7]	–	–	
Antimycotic drug used	75 (0%)	–	–	–	–	
–Posaconazole		–	73 (98%)	–	–	
–Isavuconazole		–	1(1%)	–	–	
–Caspofungin		–	1(1%)	–	–	
**Laboratory values**						
Lactate (mmol/l)	127 (4%)	1.2 [0.9–1.9]	1.2 [0.9–1.9]	1.4 [0.9–1.9]	0.980	0.462
IL-6 (pg/ml)	114 (14%)	83 [28–258]	76 [28–185]	89 [29–258]	0.448	0.907
CRP (mg/l)	130 (2%)	112 [70–177]	113 [76–196]	112[58–151]	0.237	0.573
Ferritin (ng/ml)	113 (13%)	1334 [635–2196]	1459 [755–2164]	1128 [493–2306]	0.361	0.240
hs-TnT (pg/ml)	119 (10%)	24 [11–61]	22 [11–58]	27 [11–92]	0.488	0.889
D-Dimer (mg/l)	121 (8%)	1.8 [0.9–4.7]	1.5 [0.9–3.4]	2.4 [1.1–7.7]	0.134	0.343
Creatine (mg/dl)	132 (0%)	1.1 [0.81–1.42]	1.0 [0.8–1.4]	1.2 [0.8–1.7]	0.139	0.998
Bilirubin total (mg/dl)	132 (0%)	0.5 [0.4–0.7]	0.5 [0.4–0.7]	0.5 [0.4–0.7]	0.774	0.784
SARS-CoV-2-Antibody status at ICU admission	132 (0%)	–	–	–	0.612	0.617
–positive		35 (27%)	20 (27%)	15 (26%)	–	–
–negative		33 (25%)	21 (27%)	12 (21%)	–	–
–not available		64 (48%)	34 (46%)	30 (52%)	–	–
**Blood counts**						
Leukocytes [G/l]	132 (0%)	9.4 [6.8–13.3]	9.4 [7.0–13.4]	9.2 [6.4–13.3]	0.646	0.606
Neutrophils [G/l]	132 (0%)	8.4 [6.0–11.6]	8.3 [6.0–11.6]	9.2 [6.0–11.9]	0.834	0.322
Lymphocytes [G/l]	131 (1%)	0.7 [0.5–0.9]	0.8 [0.5–1.0]	0.6 [0.5–0.9]	0.299	0.340
Thrombocytes [G/l]	132 (0%)	221 [158–309]	240 [161–335]	208 [152–293]	0.164	0.834
**Specific Medication**						
Glucocorticoids §	132 (0%)	132 (100%)	75 (100%)	57 (100%)	1.000	1.000
Remdesivir	132 (0%)	53 (40%)	36 (48%)	17 (30%)	0.035	0.555
Tocilizumab	132 (0%)	1 (1%)	1 (1%)	0 (0%)	0.382	0.326
Convalescent Plasma	132 (0%)	50 (38%)	32 (43%)	18 (32%)	0.193	0.871
**Outcomes**						
CAPA	132 (0%)				0.008	0.015
–probable CAPA	–	9 (7%)	1 (1%)	8 (14%)		
–possible CAPA	–	1 (1%)	0 (0%)	1 (2%)		
Deceased at data cut off	132 (0%)	65 (49%)	39 (52%)	26 (46%)	0.467	–
Length of ICU stay	132 (0%)	13 [5–25]	13 [6–24]	12 [4–25]	0.700	–

Data are reported as medians [25th‐75th percentile] or as absolute counts (%)

*p* denotes *p* values before ITPW weighting, *p*_IPTW_ denotes *p* values after IPTW adjustment

*p* values are either from rank‐sum tests, χ^2^‐tests, or Fisher's exact tests, as appropriate

The discrepancy between missing variable regarding pO_2_/FiO_2_ and ARDS classification inspired by the Berlin 2015 classification arises from missing exact pO_2_/FiO_2_ values; however, ARDS was classified

Abbreviations: BMI body mass index; ICU intensive care unit; COPD chronic obstructive pulmonary disease; SOFA sequential organ failure assessment; PEEP positive end expiratory pressure; vvECMO veno-venous extracorporeal membrane oxygenation, NIV non-invasive ventilation, HFNC high flow nasal cannula; IL-6 interleukin 6; CRP C- reactive protein; hs-TnT high sensitive troponin T, SARS-CoV2 severe acute respiratory syndrome corona virus 2; CAPA coronavirus disease-associated pulmonary aspergillosis

^$^ documented coronary heart disease either by specific coronary imaging or coronary angiography

^†^ comprises immunosuppressive medication (low dose of glucocorticoids are excluded) as well as diseases with severe immunosuppression

^§^ Glucocorticoids included low-dose dexamethasone or equivalent doses of other glucocorticoids

### Mould active antifungal prophylaxis

Out of 75 patients receiving antifungal prophylaxis, 73 (98%) received standard dosage of intravenous posaconazole as mould active antifungal prophylaxis as recommended by our in-house protocol. One patient (1%) received intravenous isavuconazole due to impaired tolerance against posaconazole. One patient (1%) received caspofungin due to a suspected prior anaphylactic reaction against azoles. All patients within the prophylaxis group were started on antifungal prophylaxis within 48-h after ICU admission (Table 1). At both ICU´s routine serum-galactomannan and bronchoalveolar lavage-galactomannan testing was performed, and no differences in the frequencies of GM testing was observed between the prophylaxis and no prophylaxis group excluding any observation bias (Additional file 3: Table S1).

### Propensity score

As outlined in Table 1, patients in the prophylaxis group had a lower body mass index, higher rates of chronic kidney disease, higher serum levels of creatinine and haemodialysis, lower rates of coronary artery disease, a higher proportion of immunosuppression, lower sequential organ failure score (SOFA), less frequencies in mask oxygen supply, lower levels of D-Dimer at ICU admission and received more frequently remdesivir as treatment for COVID-19 (Additional file 4: Figure S3). These imbalances between the treatment groups might display a non-random assignment bias. We pre-specified a propensity score model including a maximum of 10 variable; however, only 9 met the criteria of *p* ≤ 0.16 and/or SMD ≥ 0.31. Therefore, we predicted a propensity score based on a 9-variable multivariable logistic regression model (Additional file 5: Table S2) to control for the between group differences. The propensity score covered the whole probability range (Additional file 6: Figure S4A) and was then transformed into the IPTW (Additional file 6: Figure S4B). The propensity score was able to eliminate all difference between the treatment groups after re-weighting of the data (Table 1, Additional file 4: Figure S3) which allowed us to correct for a potential non-random assignment bias and provide more reliable results. Moreover, the trimmed IPTW showed the same potential in reducing the between group differences showing a high reliability of the computed propensity score (data not shown).

### CAPA incidence and mould active antifungal prophylaxis

During the first 30-days after ICU admission, we observed 10 CAPA cases (9 probable and 1 possible CAPA cases), corresponding to 1-, 15-, and 30-day CAPA incidence of 0.8% (95% CI 0.1–5.2), 8.5% (4.6–15.2), and 8.5% (4.6–15.2), respectively. No case of CAPA was diagnosed after 30-days of ICU admission. CAPA cases were diagnosed after a median 6 days following ICU admission (25th–75th percentile 3–9) and presented with positive BAL galactomannan (GM) > 1.0 optical density index (ODI, 7/10), BAL culture growing *Aspergillus* species (4/10), positive serum GM > 0.5 ODI (4/10), and positive BAL *Aspergillus* PCR (5/10) in addition to the other required parameters defining CAPA. All patients analysed had at least on negative diagnostic test for CAPA according to the ECMM/ ISHAM consensus after ICU admission to exclude potential pre-emptive treatment. Patients diagnosed with CAPA were treated according the ECMM/ISHAM consensus. In detail, in the non-prophylaxis group 3 patients received isavuconazole and 6 received posaconazole, the patient in the prophylaxis group developing a breakthrough infection was switched to isavuconazole. Detailed characteristics of the CAPA patients are summarized in Additional file 7: Table S3.

**Fig. 2:**
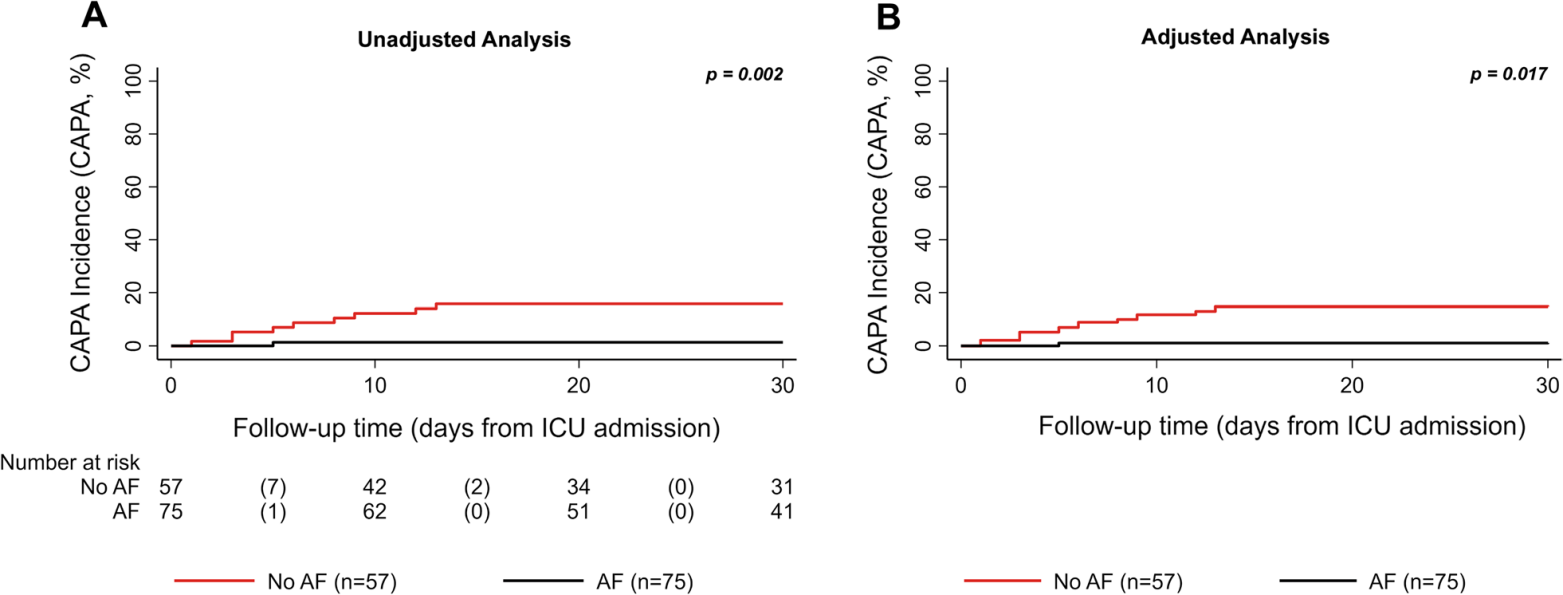
CAPA incidence according to antifungal prophylaxis. 30-day CAPA incidence in patients without (red curve) and with (black curve) mould active antifungal prophylaxis. A competing risk analysis was performed as death is a competing risk for development of CAPA. Panel **A** denotes the unadjusted analysis (17.5 vs 1.4%, *p* = 0.002). Panel **B** denoted the IPTW weighted (adjusted) analysis (15.8 vs 1.1%, *p* = 0.002). Abbreviation: AF antifungal prophylaxis; CAPA COVID-19-associated pulmonary aspergillosis; ICU intensive care unit

Of the 10 CAPA cases, 9 occurred in patients not receiving anti-mould prophylaxis, while one occurred in the anti-mould prophylaxis group (one time serum GM positivity as only mycological criterion in that patient, while BAL GM was negative). With the unadjusted competing risk analysis, the 30-day CAPA incidence estimates were 17.5% (95% CI 9.6–31.4) in the non-antifungal prophylaxis group and 1.4% (95% CI 0.2–9.7) in those receiving anti-mould prophylaxis (Gray´s test *p* = 0.002, Fig. 2A). In the Fine and Gray´s model, this corresponded to a sub-distributional hazard Ratio (SHR) of 0.08 (95% CI 0.01–0.63, *p* = 0.017) in the group with versus without administration of mould active antifungal prophylaxis.

In addition, we performed an analysis using the re-weighted data with the generated IPTW. After adjustment, the 30-day CAPA incidence estimates were 15.8% in the non-antifungal prophylaxis group and 1.1% in the mould active antifungal prophylaxis group (Gray´s test *p* = 0.001, Fig. 2B). In the Fine and Gray´s model, this corresponded to a SHR of 0.07 (95% CI 0.01–0.57, *p* = 0.013) for administration of mould active antifungal prophylaxis.

### Univariable and multivariable predictors of CAPA

Further univariable analyses of 30-day-CAPA identified higher positive end-expiratory pressure (HR per 1 cmH_2_O increase = 1.04, 95% CI 1.02–1.07, *p* = *0.001*), endotracheal intubation (HR 4.89, 95% CI 1.03–23.03, *p* = 0.045), and decreased absolute lymphocyte counts (HR per 1 G/l increase = 0.30, 95% CI 0.10–0.93, *p* = 0.037) as factors associated with CAPA (Additional file 8: Table S4). To determine the independent prognostic value of mould active antifungal prophylaxis for 30-day-CAPA development, multivariable analyses including all univariable predictors were performed. Here, the prognostic association prevailed. (Additional file 9: Table S5).

### Mould active antifungal prophylaxis, CAPA, and ICU survival

Within 30 days of ICU admission, 48 deaths were observed, corresponding to 1-, 15-, and 30-day ICU survival estimates of 96% (95% CI 91–98), 78% (70–84), and 63% (54–71), respectively, in the whole cohort (Additional file 2: Fig. S2).

**Fig. 3:**
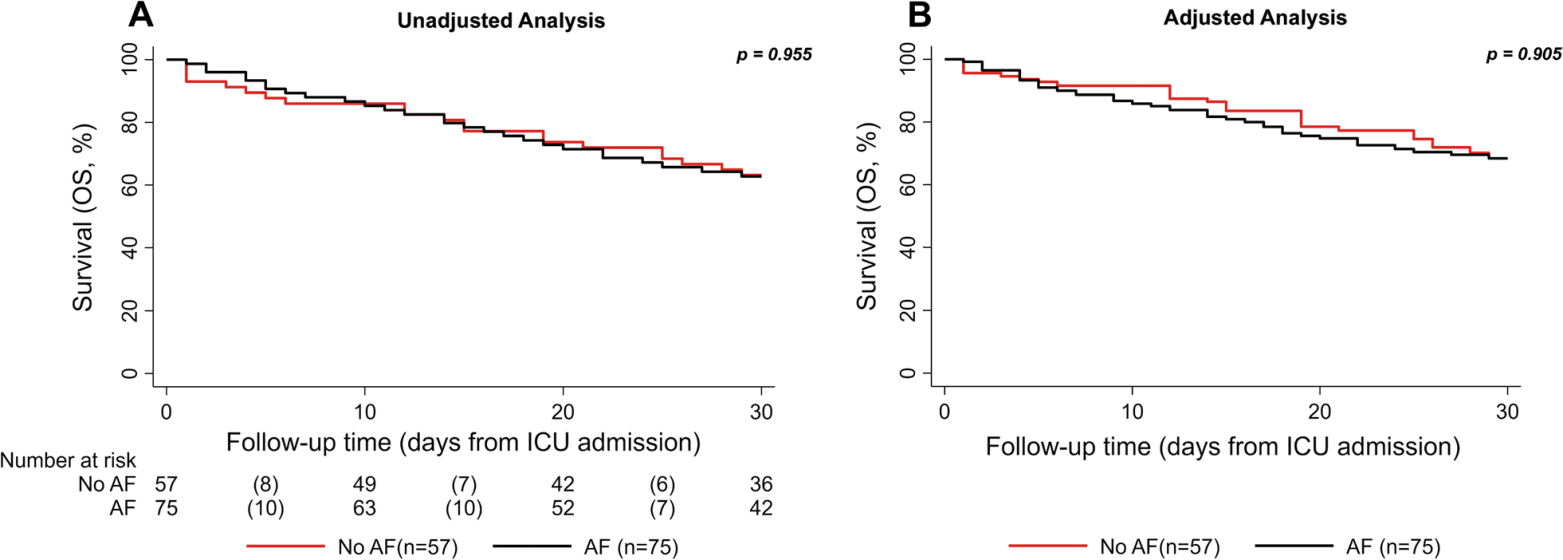
30-day ICU survival according to antifungal prophylaxis. **A** Unadjusted analysis, **B** IPTW adjusted analysis. *p* values are calculated using the log rank test. Risk table was only computed for the unadjusted analysis. ICU survival was calculated by using Kaplan–Maier estimators. CAPA coronavirus disease 19-associated pulmonary aspergillosis; AF antifungal prophylaxis

In the time to event analysis, patients receiving antifungal prophylaxis experienced no improvement of 30-day ICU survival outcomes: 1-, 15- and 30-day ICU survival rates were 98.6% (95% CI 90.1–99.8), 78.3% (67.1–86.2) and 62.7% (50.5–72.6) in patients given mould active antifungal prophylaxis, and 93.0% (82.3–97.3), 77.1% (64.0–86.1) and 63.1% (49.2–74.1) in those not receiving mould active prophylaxis, respectively. The Kaplan–Meier analysis results revealed no differences between the treatment groups (log-rank *p* = 0.955) (Fig. 3A). To account for the non-random assignment bias, we also re-weighted this data for the IPTW. However, the re-weighted analysis also showed no differences in 30-day ICU survival between patients receiving antifungal prophylaxis and those who did not (log-rank *p* = 0.905) (Fig. 3B). To investigate a potential effect of mould-active antifungal prophylaxis on long-term ICU survival, we repeated the time-to-event analysis for 90-day ICU survival. Interestingly, no difference between both groups could be observed regarding this co-secondary endpoint (Additional file 10: Fig. S5).

**Fig. 4:**
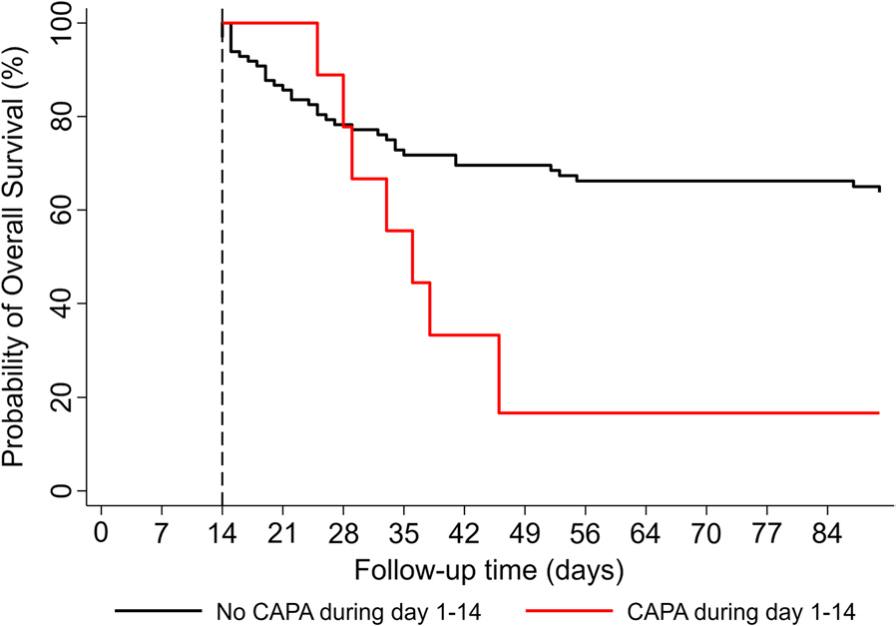
Landmark analysis of 90-day ICU survival according to CAPA diagnosis. Day 14 after ICU admission was chosen as landmark date, as most CAPA diagnosis occurred before this time point. 90-day overall survival estimates were 52.9% in patients without CAPA and 14.6% in patients with CAPA (*p* = 0.026). Abbreviation: CAPA COVID-19-associated pulmonary aspergillosis; ICU intensive care unit

Using a univariable time-to-event regression analysis treating development of CAPA as dependent variable, we also evaluated impact of CAPA on 90-day ICU survival as co-secondary outcome for long-term survival. Patients who developed CAPA during their ICU stay displayed worse outcomes regarding 90-day ICU mortality (HR 2.30, 95% CI 1.08–4.91, *p* = 0.031). These findings prevailed in multivariable regression analyses adjusting for prognostic co-variates, such as age, SOFA, creatinine, number of comorbidities, body-mass-index, immunosuppression, and supportive treatment with convalescent plasma as previously described (Additional file 11: Table S6). In a landmark analysis after 14 days, 90-day ICU survival estimates were 63.8% and 16.7% in patients in patients with and without CAPA during their ICU stay (Mantel–Byar *p* = 0.026, Fig. 4).

## Discussion

We observed that mould-active antifungal prophylaxis for patients with COVID-19-associated acute respiratory failure admitted to the ICU was highly effective in preventing CAPA. In patients without antifungal prophylaxis, CAPA was diagnosed in 17.5% versus only in 1.4% of those receiving prophylaxis, representing a reduction in relative risk for development of CAPA of 92% in the prophylaxis group and a number needed to receive prophylaxis of 7 to prevent one CAPA case. These results outline the efficacy of prophylaxis with posaconazole. While CAPA was associated with significantly higher mortality, antifungal prophylaxis did not have a significant impact on overall survival.

In our study, mould active antifungal prophylaxis resulted in a > 90% reduction of CAPA, outlining the effectiveness of antimould prophylaxis in this setting. After adjusting for differences in baseline characteristics, the positive effect of mould active antifungal prophylaxis was even stronger. The vast majority of patients in our study received posaconazole prophylaxis, the current gold standard for antifungal prophylaxis in patients with haematological malignancies [34, 35]. Posaconazole and particularly its newer tablet and intravenous formulations have several advantages compared to other drugs that are used for prophylaxis, starting with favourable pharmacokinetics, long half live and high intracellular concentrations [36]. Voriconazole and isavuconazole are predominantly metabolized by the CYP enzyme system resulting in higher rates of drug–drug interactions, when compared to posaconazole which is metabolized predominantly through the uridine diphosphate-glucuronyltransferase enzyme pathways [37–39]. Voriconazole is in fact among the drugs most frequently associated with major drug-drug interactions in the ICU setting [40], and may also show interactions with remdesivir which also is a substrate for CYP3A4, although its metabolism is primarily mediated by hydrolase activity [41]. Administration of intravenous liposomal Amphotericin B is complicated by the fact that SARS-CoV-2 has shown renal tropism and has been described as a frequent cause of acute kidney injury [42]. Inhaled amphotericin B may be an alternative for prophylaxis of CAPA but lacks regulatory approval in this application and has been associated with severe adverse events [43]. Therefore, posaconazole seems for now an appropriate choice for CAPA prophylaxis, although isavuconazole may be an alternative. New antifungal classes currently under development, namely fosmanogepix and olorofim, which show equal efficacy with minimal drug–drug interactions and toxicity in current phase IIb studies may become options/alternatives soon.

Using antimould prophylaxis for prevention of CAPA is attractive, because CAPA has been associated with high mortality rates [2–4, 12, 15], which was confirmed in our study where patients who developed CAPA during their ICU stay displayed worse outcomes with a more than twofold higher mortality compared to patients without CAPA. Given that patients who succumb very early after ICU admission are no longer able to develop CAPA—and most diagnoses occurred within 14 days after ICU admission in our cohort (median time to diagnosis 6 days after ICU admission)—we performed a landmark analysis from this time point and found that survival estimates were 64% in patients without CAPA compared to 17% in those diagnosed with CAPA. While early treatment may reduce mortality [2], early diagnosis of CAPA is challenging because clinical presentation and imaging findings of COVID-19 and CAPA may overlap (fever, shortness of breath, cough; unspecific infiltrates and consolidations, halo sign), and serum galactomannan has a low sensitivity [1, 15]. While prolonged neutropenia, stem-cell transplantation, immunosuppression, underlying lung disease, or systemic corticosteroids have been described as risk factors for development of CAPA [1], none of those is a strong predictor of CAPA, which may very well also develop in patients without any other risk factors than COVID-19-associated acute respiratory failure [1].

Pulmonary aspergillosis may also complicate acute respiratory failure caused by other viral infections, among those with severe influenza where we have recently observed three cases of pulmonary aspergillosis in our centre [19].

In a recent study on anti-mould prophylaxis to prevent influenza-associated aspergillosis [44], posaconazole did not show significant benefit due to the fact that patients developed invasive aspergillosis mostly within 48 h of ICU admission (i.e. before posaconazole reaches its steady state [45]). In contrast, CAPA has been shown before to develop mostly > 5 days after ICU admission, by which time steady-state posaconazole levels are usually reached [15, 46, 47], making it a better target for antifungal prophylaxis.

Importantly, the prevalence of CAPA has been shown to vary between centres. The prevalence of 17.5% observed in our cohort not receiving anti-mould prophylaxis was higher than the median 8.9% reported previously for COVID-19 patients admitted to ICU [3, 6–11, 20, 48, 49], but has been lower than the median 20.1% reported in other studied for COVID-19 patients requiring invasive ventilation [4, 6, 7, 12–14]. Whether or not the > 90% efficacy in reducing CAPA shown in our study will justify the broad use of antimould prophylaxis in other centres will mainly depend on local prevalence rates of CAPA. Targeted use of prophylaxis in certain groups of patients at higher risk may also be an option for some centres.

Our study has several limitations including those patients were not randomized to receive mould active antifungal prophylaxis, and cases and controls were enrolled—in part—at different ICUs. However, we found similar baseline characteristics in patient with or without mould active antifungal prophylaxis, and additionally adjusted for any imbalances using a propensity score and IPTW weighted analysis. Also, this is a single-centre study and although the observed prevalence in our centre was within the range reported before, the reported range is wide and prevalence of CAPA observed in our centre may not translate to other centres.

## Conclusion

In conclusion, this is the first study evaluating efficacy of mould active antifungal prophylaxis in ICU patients suffering from acute respiratory failure due to COVID-19. We were able to show in our cohort that antifungal prophylaxis was able to prevent CAPA. The occurrence of CAPA was found to be associated with poor outcomes.

## Supplementary Information

**Additional file 1.** Statistical analysis plan

**Additional file 2.** Overall survival and CAPA incidence

**Additional file 3.** Galactomannan testing per ICU week.

**Additional file 4.** Standardized mean difference (SMD) plot

**Additional file 5.** A propensity score model for treatment group assignment

**Additional file 6.** Histograms of the Propensity Score and the IPTW.

**Additional file 7.** Characteristics of CAPA patients

**Additional file 8.** Univariable Predictors of CAPA.

**Additional file 9.** Multivariable Cox regression of overall survival according to antifungal prophylaxis in the overall cohort

**Additional file 10.** 90-day ICU survival according to antifungal prophylaxis

**Additional file 11.** A multivariable cox regression model for 90-day ICU survival for adjustment of post-event CAPA for 7 important predictors of ICU survival

## Data Availability

The blinded analysed dataset is available on request by the first author. Email: stefan.hatzl@medunigraz.at.

## Code availability

The full analysis code (STATA) is available on request by the first author. Email: stefan.hatzl@medunigraz.at.
