# Supplementary material for: Antifungal prophylaxis for prevention of COVID-19-associated pulmonary aspergillosis in critically ill patients: an observational study
Source: Crit Care. 2021 Sep 15;25:335. doi: 10.1186/s13054-021-03753-9 (PMC8441945; doi:10.1186/s13054-021-03753-9)
Supplement: Supplementary file 1 — Additional file 1. Statistical analysis plan [file 13054_2021_3753_MOESM1_ESM.docx]

| **Variable** | **90-day ICU survival** |
| --- | --- |
|  | **HR (95%CI, p)** |
|  |  |
| CAPA | 2.28 (1.01-5.22, **p=0.049**) |
| Age per year | 1.03 (1.01-1.06, **p=0.001**) |
| SOFA per point | 1.26 (1.09-1.45, **p=0.002**) |
| Creatine per mg/dl | 0.92 (0.79-1.07, **p=0.295**) |
| Number of conditions | 1.16 (1.01-1.32, **p=0.028**) |
| BMI | 1.02 (0.98-1.07, **p=0.225**) |
| Immunosuppression | 2.50 (0.98-1.07, **p=0.225**) |
| Convalescent plasma | 1.36 (0.75-2.45, **p=0.306**) |

**Supplementary Table 5:** A multivariable cox regression model for 90-day ICU survival for adjustment of post-event CAPA for 7 important predictors of ICU survival

CAPA – corona virus disease 19 associated pulmonary aspergillosis; SOFA-sequential organ failure assessment; BMI – body mass index
