# Supplementary material for: Antifungal prophylaxis for prevention of COVID-19-associated pulmonary aspergillosis in critically ill patients: an observational study
Source: Crit Care. 2021 Sep 15;25:335. doi: 10.1186/s13054-021-03753-9 (PMC8441945; doi:10.1186/s13054-021-03753-9)
Supplement: Supplementary file 2 — Additional file 2. Overall survival and CAPA incidence [file 13054_2021_3753_MOESM2_ESM.docx]

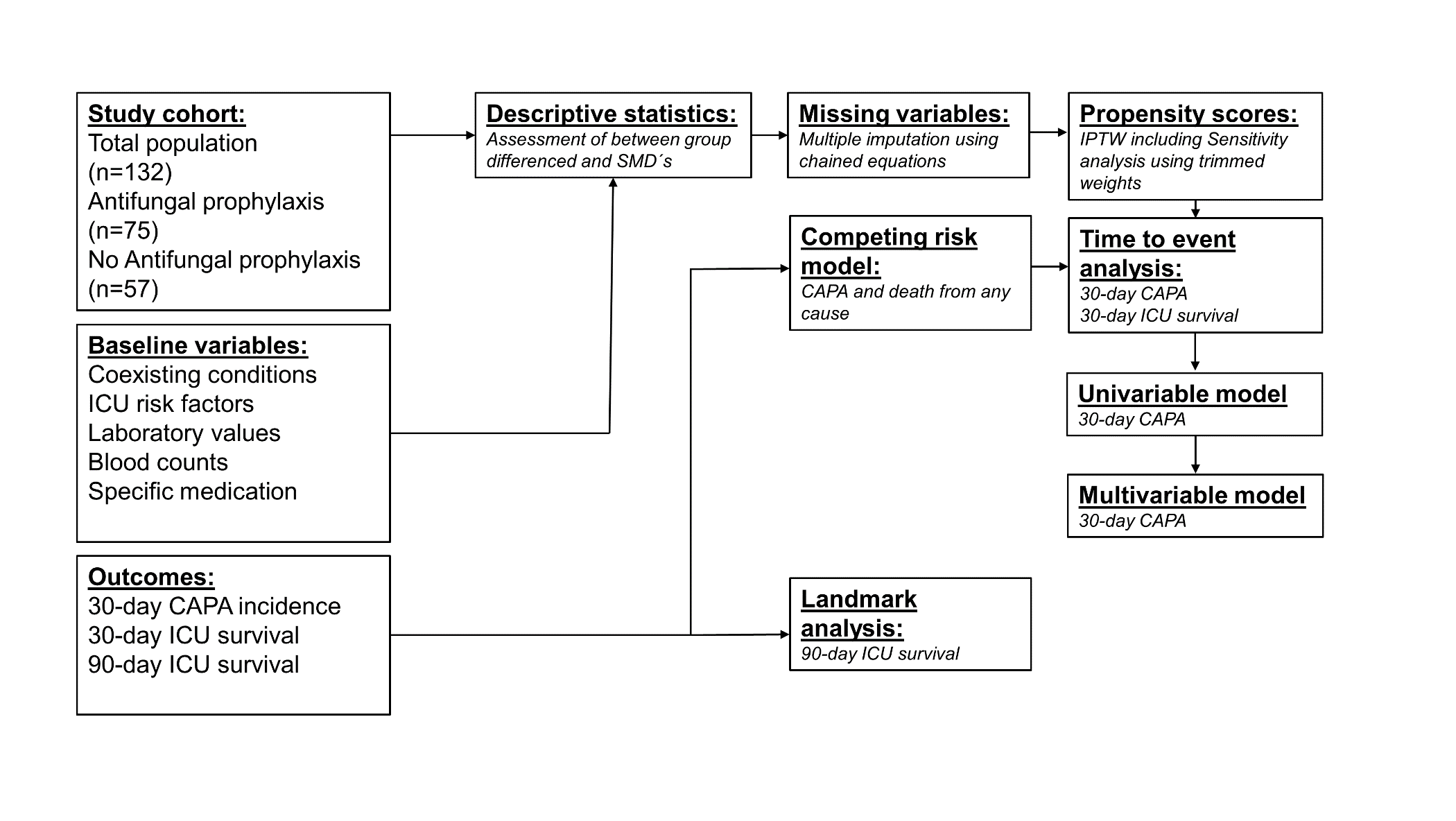


**Supplementary Figure 1:** Statistical analysis plan.

CAPA- COVID-19 associated pulmonary aspergillosis; ICU- intensive care unit; SMD – standardized mean difference; IPTW – invers probability of treatment weight; SW – stabilized weights;
