# Supplementary material for: Antifungal prophylaxis for prevention of COVID-19-associated pulmonary aspergillosis in critically ill patients: an observational study
Source: Crit Care. 2021 Sep 15;25:335. doi: 10.1186/s13054-021-03753-9 (PMC8441945; doi:10.1186/s13054-021-03753-9)
Supplement: Supplementary file 3 — Additional file 3. Galactomannan testing per ICU week. [file 13054_2021_3753_MOESM3_ESM.docx]

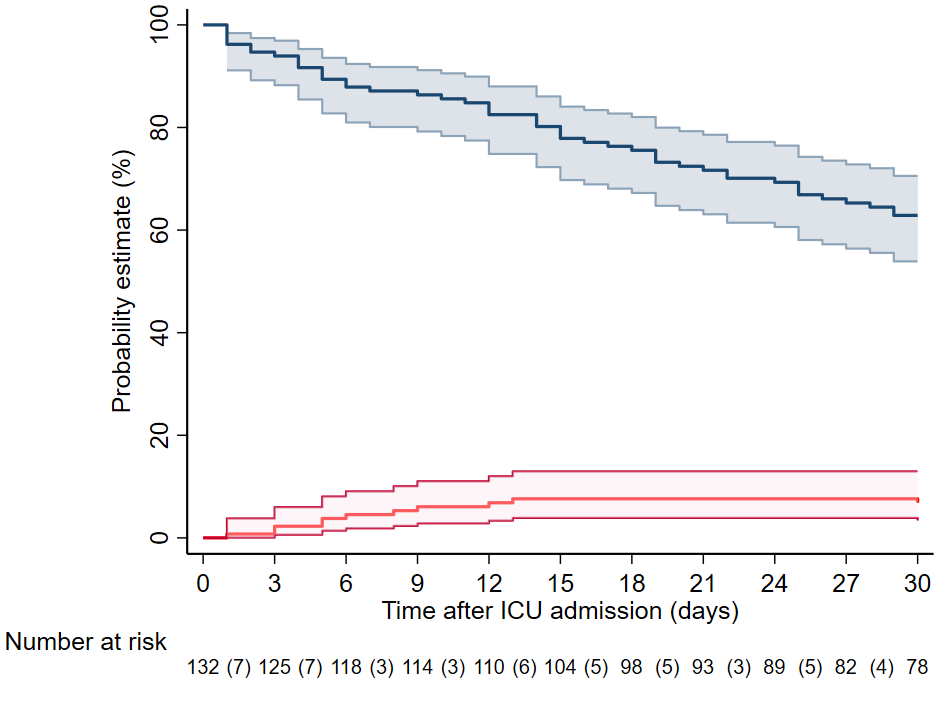


**Supplementary Figure 2:** Overall survival and CAPA incidence displayed as competing events of the whole cohort (n=132) 30-day survival estimates (63% [54-71], blue curve) and CAPA incidence estimate (8.5% [4.6-15.2], red curve) of the total cohort. Abbreviation: ICU-intensive care unit.
