# Supplementary material for: Antifungal prophylaxis for prevention of COVID-19-associated pulmonary aspergillosis in critically ill patients: an observational study
Source: Crit Care. 2021 Sep 15;25:335. doi: 10.1186/s13054-021-03753-9 (PMC8441945; doi:10.1186/s13054-021-03753-9)
Supplement: Supplementary file 4 — Additional file 4. Standardized mean difference (SMD) plot [file 13054_2021_3753_MOESM4_ESM.docx]

| **Variable** |  | **n (%miss)** | **Overall**  **(n=132)** | **Antifungal Prophylaxis**  **(n=75)** | **No Antifungal Prophylaxis**  **(n=57)** | ***p*** |
| --- | --- | --- | --- | --- | --- | --- |
|  |  |  |  |  |  |  |
| ICU weeks |  | 132 (0%) | 1.7 [0.7-3.6] | 1.9 [0.9-3.4] | 1.7 [0.6-3.6] | 0.697 |
| Serum-GM per ICU week |  | 132 (0%) | 0.6 [0.0-1.3] | 0.6 [0.0-1.3] | 0.5 [0.0-1.7] | 0.813 |
| BAL-GM per ICU week |  | 132 (0%) | 0.0 [0.0-0.3] | 0.0 [0.0-0.3] | 0.0 [0.0-0.2] | 0.293 |

**Supplementary Table 1**: Galactomannan testing per ICU week.

No differences in testing frequencies could be observed between the groups. P values were calculated via rank-sum tests.

GM = galactomannan BAL = broncho-alveolar-lavage, ICU = intensive care unit
