# Supplementary material for: Antifungal prophylaxis for prevention of COVID-19-associated pulmonary aspergillosis in critically ill patients: an observational study
Source: Crit Care. 2021 Sep 15;25:335. doi: 10.1186/s13054-021-03753-9 (PMC8441945; doi:10.1186/s13054-021-03753-9)
Supplement: Supplementary file 5 — Additional file 5. A propensity score model for treatment group assignment [file 13054_2021_3753_MOESM5_ESM.docx]

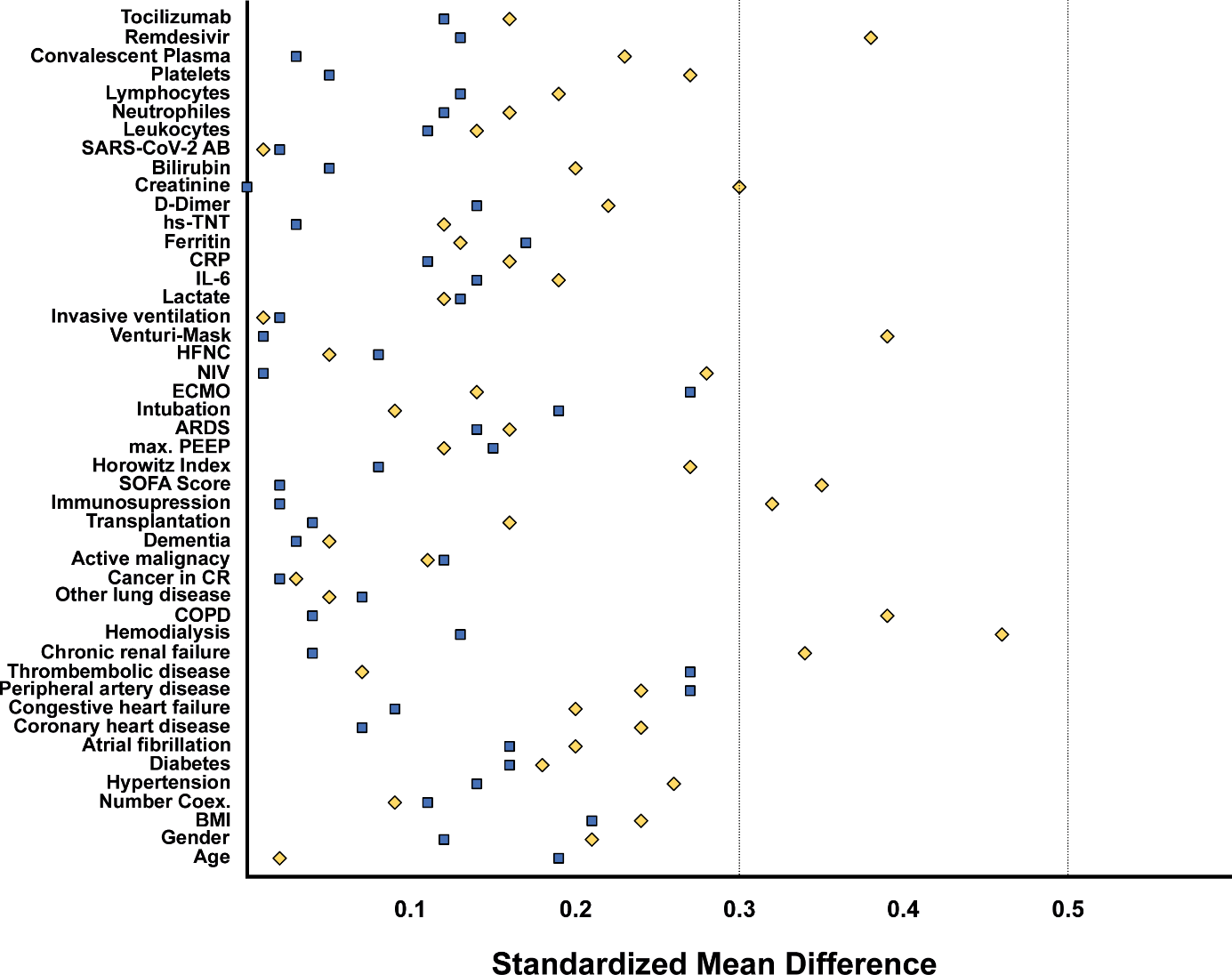


**Supplementary Figure 3:** Standardized mean difference (SMD) plot. Blue squares denote Standardized mean differences (SMDs) in before weighting the inverse of the probability of treatment weight (IPTW). Yellow diamonds denote the SMDs after weighting the IPTW. The constructed propensity score was able to balance all variables below SMD of 0.3 resulting no significant differences in between the treatment groups.

BMI – body mass index; Number Coex. – number of coexisting conditions; COPD – chronic obstructive pulmonary disease; SOFA – sequential organ failure assessment score at ICU admission; max.PEEP – maximum positive end-expiratory pressure; ARDS – acute respiratory failure classification inspired by Berlin 2015; ECMO – extra corporeal membrane oxygenation; NIV – non-invasive ventilation; IL-6 – interleukin-6; CRP – C reactive protein; hs-TNT – high sensitive Troponin T; SARS-CoV-2 AB; positivity for anti-SARS-CoV-2 antibodies at ICU admission; CR = complete remission.
