# Supplementary material for: Antifungal prophylaxis for prevention of COVID-19-associated pulmonary aspergillosis in critically ill patients: an observational study
Source: Crit Care. 2021 Sep 15;25:335. doi: 10.1186/s13054-021-03753-9 (PMC8441945; doi:10.1186/s13054-021-03753-9)
Supplement: Supplementary file 6 — Additional file 6. Histograms of the Propensity Score and the IPTW. [file 13054_2021_3753_MOESM6_ESM.docx]

| **Variable** |  | **Multivariable Odds Ratio (OR)** | **95%CI** | **P** |
| --- | --- | --- | --- | --- |
|  |  |  |  |  |
| **Demographic variables** |  |  |  |  |
| BMI (per 1 point increase) |  | 0.92 | 0.84-0.99 | 0.006 |
| Coronary artery disease |  | 0.17 | 1.31-2.48 | 0.038 |
| Dialysis |  | 0.16 | 0.02-1.59 | 0.117 |
| COPD |  | 6.68 | 1.29-34.59 | 0.024 |
| Creatine (per 1 unit increase) |  | 1.17 | 0.81-1.68 | 0.405 |
| D-Dimer (per 1 unit increase) |  | 0.98 | 0.94-1.02 | 0.478 |
| SOFA-score (per 1 point increase) |  | 0.85 | 0.66-1.01 | 0.193 |
| Mask oxygen supply |  | 0.09 | 0.01-2.47 | 0.157 |
| Immunosuppression |  | 3.06 | 0.73-12.77 | 0.125 |
| Remdesivir |  | 2.12 | 0.87-5.15 | 0.063 |

**Supplementary Table 2:** A propensity score model for treatment group assignment

We predicted a 9-variable propensity score model, due to the collinearity of dialysis, creatinine, and chronic kidney disease we omitted choric kidney disease from the final model.

BMI- body mass index; COPD -chronic obstructive pulmonary disease; SOFA -sequential organ failure assessment.
