# Supplementary material for: Antifungal prophylaxis for prevention of COVID-19-associated pulmonary aspergillosis in critically ill patients: an observational study
Source: Crit Care. 2021 Sep 15;25:335. doi: 10.1186/s13054-021-03753-9 (PMC8441945; doi:10.1186/s13054-021-03753-9)
Supplement: Supplementary file 7 — Additional file 7. Characteristics of CAPA patients [file 13054_2021_3753_MOESM7_ESM.docx]

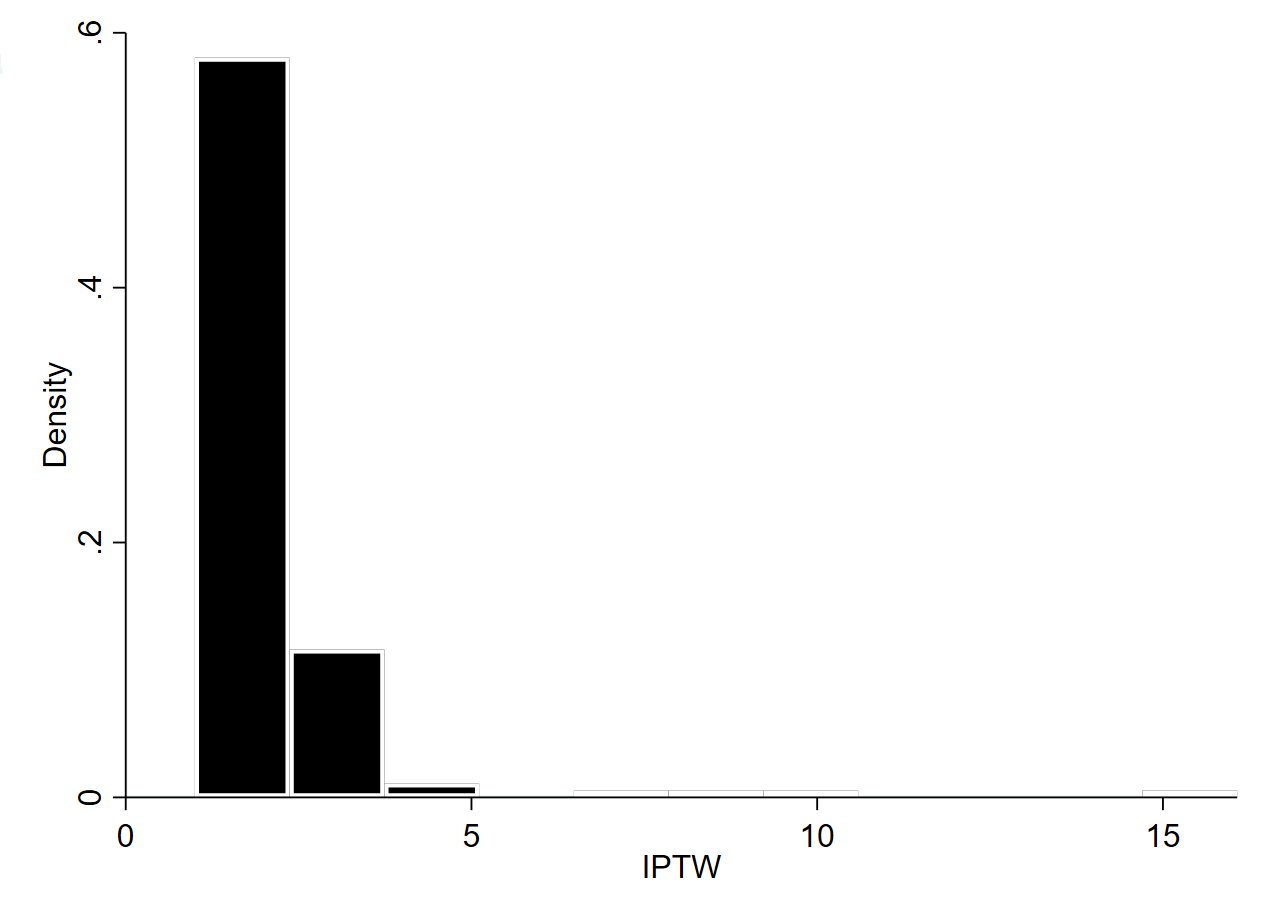

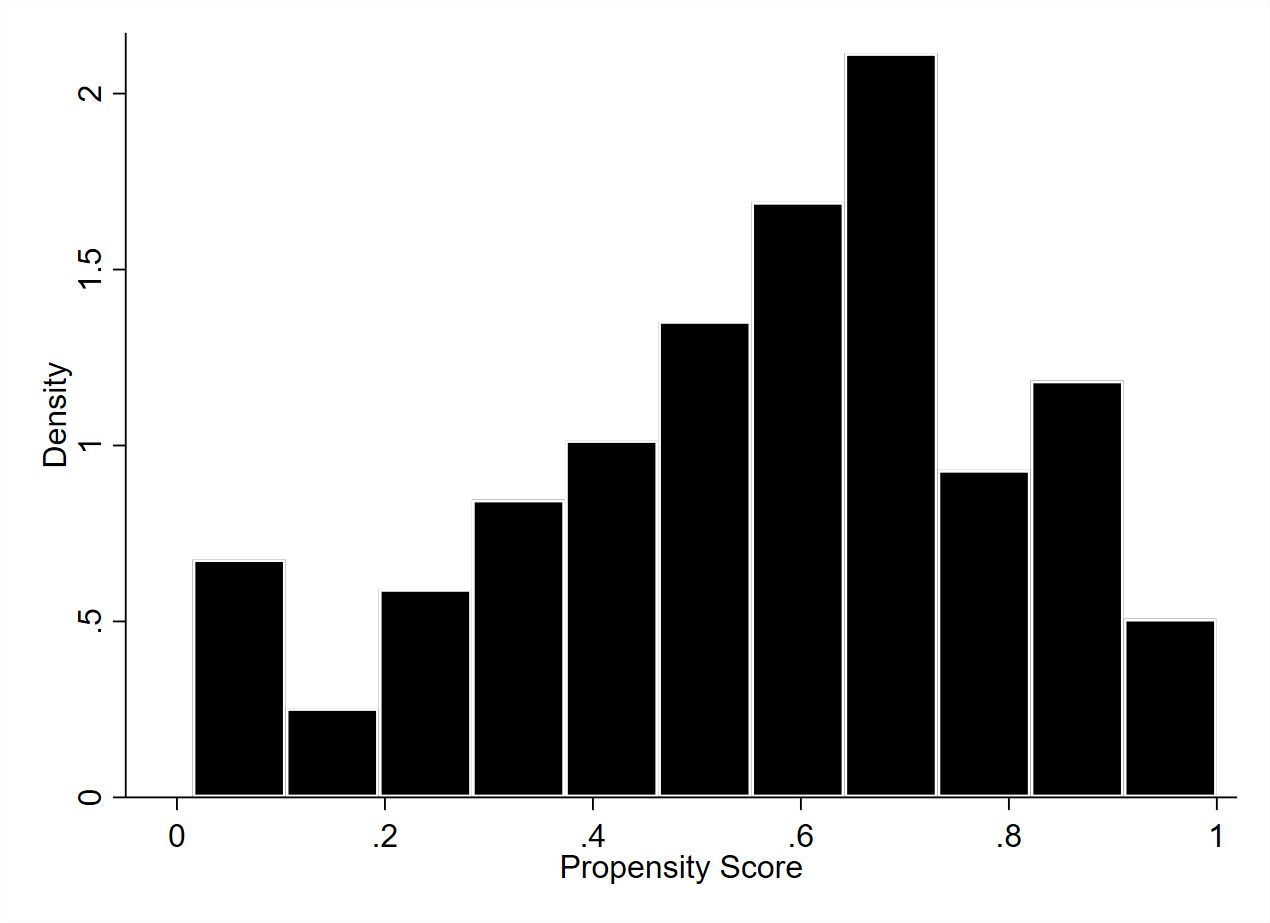


**A**

**B**

**Supplementary Figure 4:** Histograms of the Propensity Score and the IPTW. (A) The propensity score can range from 0 to 1. Multiply by 100 to obtain probabilities (in percent) of having received mold active antifungal prophylaxis. (B) The IPTW was defined as the inverse of the probability of receiving the treatment that the patient received
