# Supplementary material for: Antifungal prophylaxis for prevention of COVID-19-associated pulmonary aspergillosis in critically ill patients: an observational study
Source: Crit Care. 2021 Sep 15;25:335. doi: 10.1186/s13054-021-03753-9 (PMC8441945; doi:10.1186/s13054-021-03753-9)
Supplement: Supplementary file 8 — Additional file 8. Univariable Predictors of CAPA. [file 13054_2021_3753_MOESM8_ESM.docx]

| **Patient Identifier** | **Group** | **Ventilation form** | **paO_2_/FiO_2_** | **Days to first test for CAPA** | **Type of first test of CAPA** | **Days to first positive test** | **Type of first positive test** | **Treatment of CAPA** | **Outcome** |
| --- | --- | --- | --- | --- | --- | --- | --- | --- | --- |
| UPN 18 | No-AF | Invasive | 68 | 1 | Serum-GM | 13 | BAL-GM/ | Posaconazol | Deceased |
| UPN 31 | No-AF | Invasive | 92 | 0 | Serum/BAL-GM | 9 | Asp-PCR | Isavuconazol | Deceased |
| UPN 44 | No-AF | Invasive | 75 | 0 | Serum-GM | 12 | BAL-GM/ Asp-PCR | Isavuconazol | Deceased |
| UPN 45 | No-AF | Invasive | 76 | 1 | Serum-GM | 5 | Culture-BAL/ Asp-PCR | Posaconazol | Deceased |
| UPN 69 | No-AF | Invasive | 74 | 2 | Serum/BAL-GM | 3 | Culture-BAL/GM-BAL | Posaconazol | Alive |
| UPN 77 | No-AF | Non-invasive | 195 | 0 | Serum-GM | 1 | Culture-BAL/GM-BAL/ Asp-PCR | Isavuconazol | Deceased |
| UPN 95 | AF | Invasive | 147 | 0 | Serum-GM | 5 | Serum-GM | Posaconazol | Deceased |
| UPN 131 | No-AF | Invasive | 79 | 1 | Serum-GM | 3 | BAL-GM | Isavuconazol | ICU admission |
| UPN 132 | No-AF | Invasive | 98 | 2 | Serum-GM | 8 | BAL-GM | Posaconazol | Deceased |
| UPN 146 | No-AF | vvECMO | 64 | 1 | Serum/BAL-GM | 6 | Culture-BAL/GM-BAL/ Asp-PCR | Posaconazol | Deceased |

**Supplementary Table 4:** Characteristics of the CAPA patients.

UPN – unified patient number; No-AF – no antifungal prophylaxis; AF – antifungal prophylaxis; CAPA – COVID-19 associated pulmonary aspergillosis; GM – galactomannan; BAL – broncho alveolar lavage; Asp – aspergillus; PCR – polymerase chain reaction; Outcome denotes the status of the patients at data cut off
