# Supplementary material for: Antifungal prophylaxis for prevention of COVID-19-associated pulmonary aspergillosis in critically ill patients: an observational study
Source: Crit Care. 2021 Sep 15;25:335. doi: 10.1186/s13054-021-03753-9 (PMC8441945; doi:10.1186/s13054-021-03753-9)
Supplement: Supplementary file 9 — Additional file 9. Multivariable Cox regression of overall survival according to antifungal prophylaxis in the overall cohort [file 13054_2021_3753_MOESM9_ESM.docx]

| **Multivariable Model** | **Variable** | **Multivariable Hazard Ratio** | **95%CI** | **p** |
| --- | --- | --- | --- | --- |
|  |  |  |  |  |
| #1 (n=111) | **Antifungal prophylaxis** | 0.08 | 0.01-0.62 | 0.016 |
|  | PEEP (per 1 cmH_2_O increase) | 1.03 | 1.01-1.06 | 0.011 |
|  |  |  |  |  |
| #2 (n=132) | **Antifungal prophylaxis** | 0.08 | 0.01-0.59 | 0.014 |
|  | Intubation | 5.16 | 1.09-24.33 | 0.038 |
|  |  |  |  |  |
| #3 (n=131) | **Antifungal prophylaxis** | 0.07 | 0.01-0.54 | 0.011 |
|  | Lymphocytes (per 1G/L increase) | 0.05 | 0.01-0.75 | 0.031 |
|  |  |  |  |  |
| #4 (n=110) | **Antifungal prophylaxis** | 0.07 | 0.01-0.54 | 0.012 |
|  | PEEP (per 1 cmH_2_O increase) | 1.04 | 1.01-1.07 | 0.019 |
|  | Intubation | 1.90 | 0.36-9.82 | 0.443 |
|  | Lymphocytes (per 1G/L increase) | 0.05 | 0.01-1.01 | 0.051 |
|  |  |  |  |  |
|  |  |  |  |  |
|  |  |  |  |  |

**Supplementary Table 4:** Multivariable Cox regression of overall survival according to antifungal prophylaxis in the overall cohort**.** n denotes the patients included in the model. Model#1 is Antifungal prophylaxis adjusted for PEEP – positive end-expiratory pressure, Model#2 is Antifungal prophylaxis adjusted for Intubation, Model#3 is Antifungal prophylaxis adjusted for lymphocyte-count, Model#4 is Antifungal prophylaxis adjusted for PEEP, Intubation, and lymphocyte-count.
