# Supplementary material for: Antifungal prophylaxis for prevention of COVID-19-associated pulmonary aspergillosis in critically ill patients: an observational study
Source: Crit Care. 2021 Sep 15;25:335. doi: 10.1186/s13054-021-03753-9 (PMC8441945; doi:10.1186/s13054-021-03753-9)
Supplement: Supplementary file 10 — Additional file 10. 90-day ICU survival according to antifungal prophylaxis [file 13054_2021_3753_MOESM10_ESM.docx]

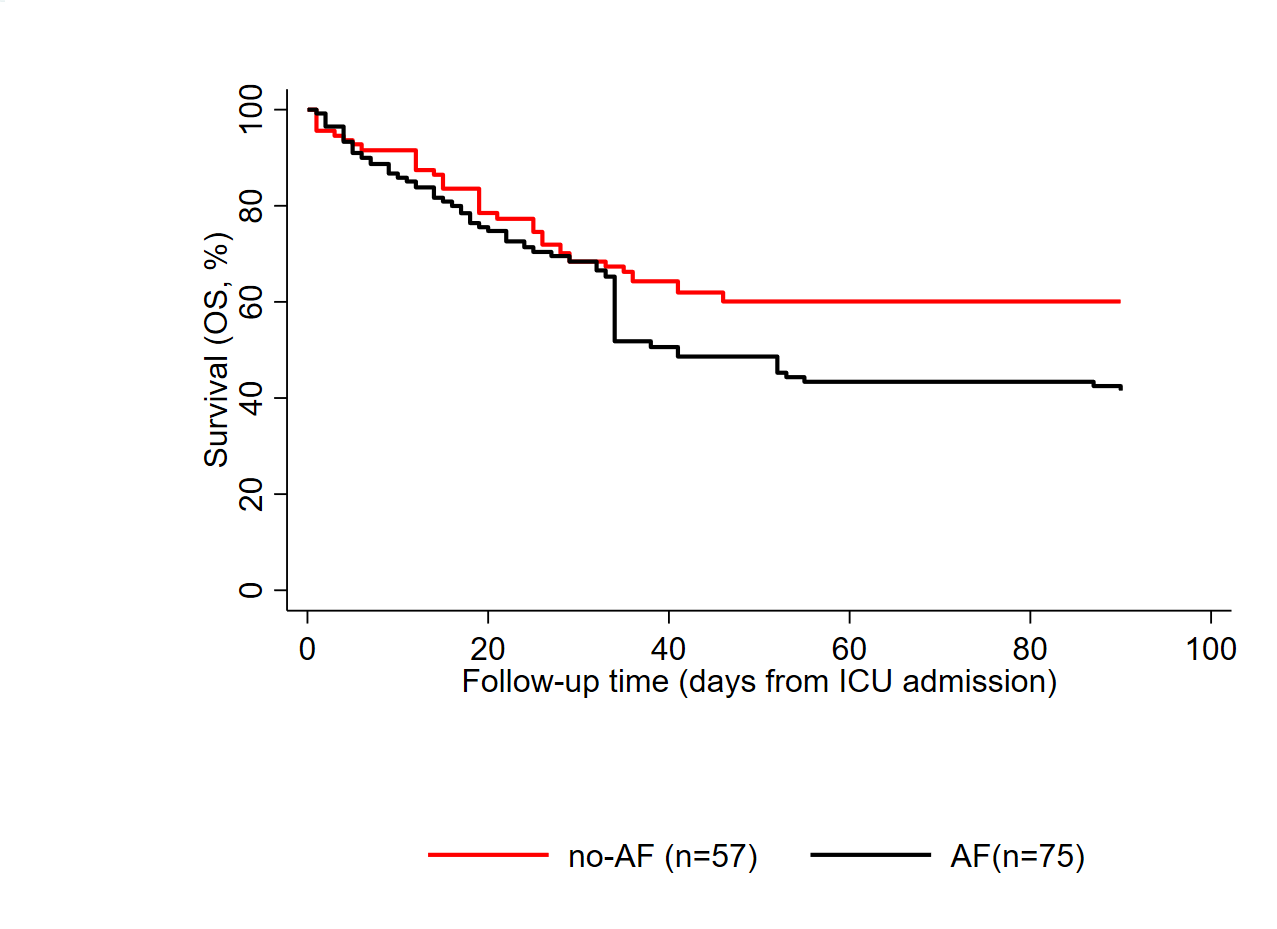

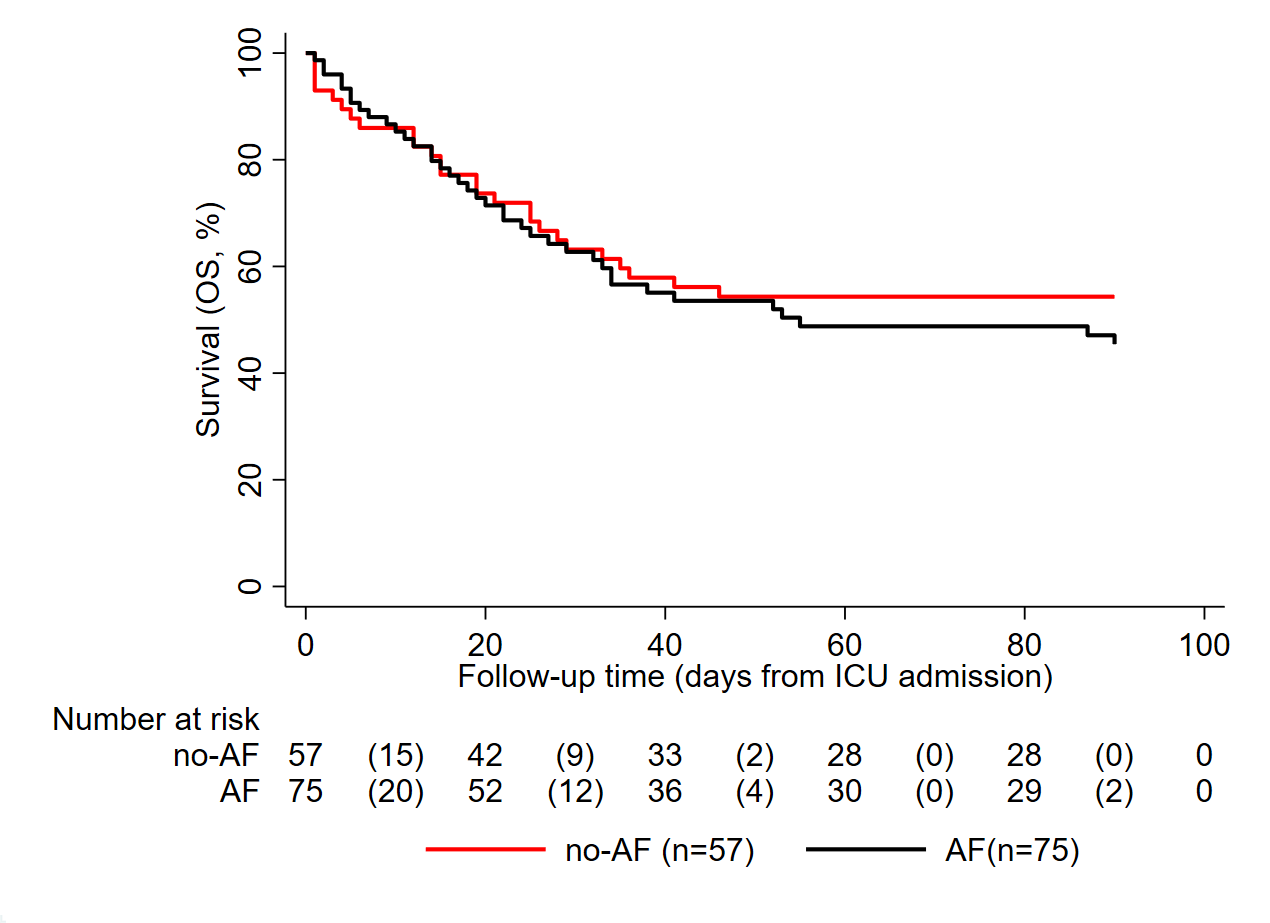


***p=0.142***

***p=0.478***

**Unadjusted Analysis**

**Adjusted Analysis**

**Supplementary Figure 5: 90-day ICU survival according to antifungal prophylaxis (Long-term survival)**

A) Unadjusted analysis B) IPTW adjusted analysis. p values are calculated using the log rank test. Risk table was only computed for the unadjusted analysis.

ICU survival was calculated by using Kaplan Maier estimators.

CAPA – coronavirus disease 19 associated pulmonary aspergillosis; AF – antifungal prophylaxis.
